# The Amyloid Plaque Proteomes of Alzheimer's Disease and Mild Cognitive Impairment

**DOI:** 10.1002/alz70855_098576

**Published:** 2025-12-23

**Authors:** Dominique Leitner, Evgeny Kanshin, Kaleah Balcomb, Manor Askenazi, Jianina Suazo, Mitchell Marta Ariza, Julie A Schneider, Beatrix Ueberheide, Eleanor Drummond, Thomas Wisniewski

**Affiliations:** ^1^ NYU Grossman School of Medicine, New York, NY, USA; ^2^ The University of Sydney, Sydney, NSW, Australia; ^3^ Biomedical Hosting LLC, Arlington, MA, USA; ^4^ Rush Alzheimer's Disease Center, Rush University Medical Center, Chicago, IL, USA; ^5^ New York University Grossman School of Medicine, New York, NY, USA

## Abstract

**Background:**

Amyloid plaques contain numerous proteins in addition to amyloid beta. Previous unbiased comprehensive localized proteomics identified plaque associated proteins in late‐onset AD (LOAD), early‐onset AD (EOAD), rapidly progressive AD (rpAD), AD in Down syndrome (DS), and preclinical AD, although with some studies having smaller cohorts and more focus on severe pathology. The amyloid plaque protein differences in mild cognitive impairment (MCI) have not been evaluated, nor how these protein differences compare to a larger AD cohort.

**Method:**

We evaluated the plaque proteomes in MCI and AD with comparisons to neighboring non‐plaque tissue and control non‐plaque tissue from ROSMAP (153 cases (*n* = 244 samples); control (*n* = 62), MCI (*n* = 36), AD (*n* = 55)). Tissue was microdissected from autopsy paraffin embedded temporal cortex and evaluated by label‐free quantitative mass spectrometry. Protein differences and complementary histology characterization of top proteins were evaluated.

**Result:**

We identified a number of differentially abundant proteins in MCI and AD plaque tissue compared to neighboring non‐plaque tissue (false discovery rate, FDR<5%). These included proteins described previously as well as novel proteins. The gene ontology (GO) terms associated with these proteins included increased inflammatory response in both MCI and AD, as well as decreased myelin in AD. Of proteins altered in at least one disease group, there were a number of proteins that were shared in MCI and AD plaque tissue vs neighboring non‐plaque. In non‐plaque tissue, increased proteins were related to GO terms DNA recombination and decreased proteins with actin‐myosin filament in AD; and there were some shared altered proteins in MCI and AD but had a mild positive correlation. Weighted gene correlation network analysis (WGCNA) evaluated how protein levels corresponded to case history and identified a number of associated proteins with regional pathology levels, overall pathology, and demographics,. After comparative analyses with other brain tissue and biofluid proteomic datasets, top protein candidates were identified and characterized further by histology.

**Conclusion:**

We have conducted the most extensive proteomic analysis of microdissected plaque proteomes in MCI and AD, with correlations to case history. Our results provide valuable insights into molecular mechanisms, novel diagnostic biomarkers, and potential therapeutic strategies.

